# Relating pre-treatment non-Gaussian intravoxel incoherent motion diffusion-weighted imaging to human papillomavirus status and response in oropharyngeal carcinoma

**DOI:** 10.1016/j.phro.2024.100574

**Published:** 2024-04-04

**Authors:** Nienke D. Sijtsema, Iris Lauwers, Gerda M. Verduijn, Mischa S. Hoogeman, Dirk H.J. Poot, Juan A. Hernandez-Tamames, Aad van der Lugt, Marta E. Capala, Steven F. Petit

**Affiliations:** a Department of Radiotherapy, Erasmus MC Cancer Institute, University Medical Center Rotterdam, Rotterdam, the Netherlands; b Department of Radiology and Nuclear Medicine, Erasmus MC, University Medical Center Rotterdam, Rotterdam, the Netherlands; c Department of Medical Physics and Informatics, HollandPTC, Delft, the Netherlands

**Keywords:** Non-Gaussian Intravoxel Incoherent Motion Imaging (NG-IVIM), Intravoxel Incoherent Motion Diffusion Kurtosis Imaging (IVIM-DKI), Diffusion-weighted imaging (DWI), Human papillomavirus (HPV), Oropharyngeal squamous cell carcinoma (OPSCC), Treatment response

## Abstract

**Background and purpose:**

Diffusion-weighted imaging (DWI) is a promising technique for response assessment in head-and-neck cancer. Recently, we optimized Non-Gaussian Intravoxel Incoherent Motion Imaging (NG-IVIM), an extension of the conventional apparent diffusion coefficient (*ADC*) model, for the head and neck. In the current study, we describe the first application in a group of patients with human papillomavirus (HPV)-positive and HPV-negative oropharyngeal squamous cell carcinoma. The aim of this study was to relate *ADC* and NG-IVIM DWI parameters to HPV status and clinical treatment response.

**Materials and methods:**

Thirty-six patients (18 HPV-positive, 18 HPV-negative) were prospectively included. Presence of progressive disease was scored within one year. The mean pre-treatment *ADC* and NG-IVIM parameters in the gross tumor volume were compared between HPV-positive and HPV-negative patients. In HPV-negative patients, *ADC* and NG-IVIM parameters were compared between patients with and without progressive disease.

**Results:**

*ADC*, the NG-IVIM diffusion coefficient *D*, and perfusion fraction *f* were significantly higher, while pseudo-diffusion coefficient D* and kurtosis *K* were significantly lower in the HPV-negative compared to HPV-positive patients. In the HPV-negative group, a significantly lower *D* was found for patients with progressive disease compared to complete responders. No relation with *ADC* was observed.

**Conclusion:**

The results of our single-center study suggest that *ADC* is related to HPV status, but not an independent response predictor. The NG-IVIM parameter *D,* however, was independently associated to response in the HPV-negative group. Noteworthy in the opposite direction as previously thought based on *ADC*.

## Highlights

• We studied ADC and NG-IVIM DWI for HPV + and HPV- oropharyngeal cancer patients.
• In our dataset, ADC is a surrogate for HPV status and not a predictor of response.
• NG-IVIM parameter D is independently related to response in HPV- patients.
• HPV status should be corrected for when assessing the predictive power of DWI.

## 1 Introduction

Diffusion-weighted imaging (DWI) is highly interesting for response assessment in head and neck (HN) cancer. Low baseline apparent diffusion coefficient (*ADC*) has been associated with favorable response to treatment compared to high baseline *ADC* [1], [2], [3], [4], [5]. Non-Gaussian Intravoxel Incoherent Motion Imaging (NG-IVIM) DWI is a novel extension of conventional DWI that enables simultaneous assessment of *inter*-cellular diffusion (similar to the *ADC* obtained from conventional DWI), but also microvascular perfusion (like IVIM DWI) and *intra*-cellular diffusion (like diffusion kurtosis imaging) [6]. Compared to conventional DWI, where only the ADC is obtained, NG-IVIM DWI provides a more detailed picture of the tumor micro-environment.

In a previous study [7], we optimized NG-IVIM DWI specifically for the HN region to allow optimal parameter estimation at maximum time efficiency*, i.e*. with a minimal number of b-values. In the current study, this optimized NG-IVIM DWI sequence was applied for the first time to a group of patients with oropharyngeal squamous cell carcinoma (OPSCC).

The current study includes two important subcategories of OPSCC patients: patients with tumors that are human papillomavirus (HPV)-negative and HPV-positive. On average, HPV-positive patients have a more favorable response to treatment than HPV-negative patients [8]. It may be important to include both categories, since a recent publication [9] suggested that the *ADC* obtained from conventional DWI might not be an *independent* prognostic factor for response, but rather a surrogate for HPV status. If that would be the case, the value of DWI for response prediction in OPSCC might be lower than expected based on earlier studies [1], [2], [3], [4], [5].

The aims of this study were to apply the optimized NG-IVIM DWI sequence for the first time in HPV-positive and HPV-negative OPSCC patients, to study differences in pre-treatment conventional DWI and NG-IVIM DWI parameters between HPV-positive and HPV-negative patients, and to relate pre-treatment conventional DWI and NG-IVIM DWI parameters to response within one year after treatment.

## 2 Materials and methods

### 2.1 Patients

This prospective study was approved by the institutional review board (protocols 20–0207 and 21–0847) and written informed consent was obtained from included patients. Eligibility criteria were OPSCC scheduled for primary (chemo-)radiotherapy; received a radiotherapy planning MRI with a multi b-value NG-IVIM DWI as part of the standard work-up between April 2020 and February 2022; and for which the primary tumor was clearly visible on the DWI image of each b-value. Tumor staging was done according to TNM classification, edition 8.

### 2.2 Treatment

Patients received either volumetric arc photon therapy or intensity-modulated proton therapy of 70 Gy (35 fractions of 2 Gy) to the primary tumor and regions containing pathological neck nodes, and 54.25 Gy to the elective neck regions, with a simultaneous integrated boost. The overall treatment time was either 7 weeks (5 fractions/week) or 6 weeks (6 fractions/week). Chemotherapy was given if indicated based on TNM stage (T3-4 or N+). Chemotherapy consisted of cisplatin (100 mg/m^2^ on days 1, 22, and 43 of treatment) or cetuximab (400 mg/m^2^ initial dose, followed by a weekly dose of 250 mg/m^2^).

### 2.3 MR imaging and post-processing

All MR imaging was performed on a 1.5 T GE MR450w (GE, Waukesha, WI, USA) using the MR Radiation Oncology Suite coils (GE, Waukesha, WI, USA) with the patient immobilized in the radiotherapy-treatment mask. The planning MRI protocol contained multi b-value DWI, a DWI scan with inverse phase encoding gradient polarity of *b* = 0 s/mm^2^ for the purpose of distortion correction [10], [11], a T2 weighted (T2w) TSE and a T1 weighted (T1w) IDEAL [12]. Gadolinium based contrast agent was administered before the start of the protocol. The multi b-value DWI scan (single-shot echo planar imaging, flip angle: 90 degrees TR: 6700 ms; TE: 81.8 ms; FOV: 26 x 26 cm; 4 mm slice thickness; 0.2 mm slice gap, 128 x 128 matrix, acceleration factor 2) consisted of 15 b-values (0, 10, 2x80, 130, 570, 2x770, 2x780, 790, and 4x1500 s/mm^2^) acquired in three orthogonal diffusion directions. These b-values are the result of a b-value optimization described in detail in previous work [7]. Distortion correction of the DWI was done with FSL topup [10], [11], based on the *b* = 0 s/mm^2^ images.

**Fig. 1:**
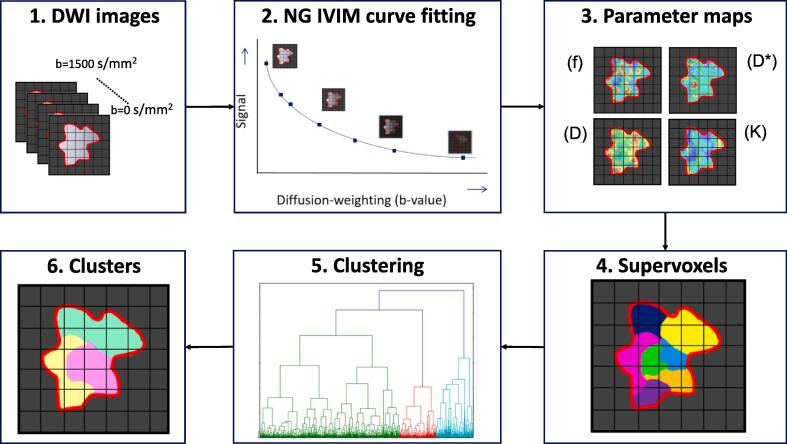
Graphical depiction of the workflow where the distortion corrected DWI images (1) are first fitted to the NG-IVIM model (2), which yields four parameters maps 3. Next, supervoxels are created based on the four parameter maps 4. Then all supervoxels from all patients are clustered (5) to gain insight in the prevalence of certain combination of parameters (identifying certain phenotypes) (6) in different tumors.

The full workflow of processing the DWI scans is depicted graphically in Fig. 1. Voxel-wise fitting was performed for the NG-IVIM and ADC model using in-house software in MATLAB (MathWorks, Natick, MA, USA). For details about the fitting procedure we refer to Supplementary Information A. Next, the average *ADC*, *f*, D*, *D*, and *K* were calculated by averaging over all gross tumor volume (GTV) voxels.

### 2.4 Gross tumor volume delineation

The GTV was delineated on the T2w images by an experienced radiation oncologist, with additional information from gadolinium enhanced T1w images. Subsequently, the T2w image was rigidly (rotation and translation) registered to the distortion corrected *b* = 0 s/mm^2^ image of the DWI scan for each patient. The contours were propagated to the DWI and manually checked on the *b* = 0 s/mm^2^ scan. If the shape of the pharynx deviated between the T2w and the *b* = 0 s/mm^2^ scan, the voxels from the GTV located in air on the *b* = 0 s/mm^2^ scan were excluded.

### 2.5 Assessing intra-tumor regions

Intra-tumor parameter heterogeneity was investigated to identify tumor regions with similar phenotypes across different patients, using unsupervised clustering. Before applying the clustering method, noise was filtered out for each GTV by creating supervoxels using the Simple Linear Iterative Clustering (SLIC) algorithm [13]. A supervoxel can be seen as a union of adjacent voxels with similar normalized NG-IVIM parameters. The SLIC algorithm automatically determines which voxels belong to which supervoxels, based on an average size set to 50 ± 10 voxels per supervoxel and a compactness set to 20.

Next, tumor regions with similar phenotypes across different patients were identified by clustering all supervoxels of all patients, using an agglomerative Ward clustering algorithm based on the average normalized parameter values of the supervoxels [14] similar to Even et al. [15]. The optimal number of clusters (i.e. regions with similar phenotypes) was selected for the entire dataset (between two and ten) based on the Calinski-Harabasz index [16]. The number of clusters with the highest Calinski-Harabasz index was selected. Per tumor, the fraction of supervoxels belonging to each cluster (*i.e*. tumor region with distinct phenotype) was calculated. This is referred to as the fractional contribution. Clustering was carried out with in-house software and the SciPy package (version 1.10.1) in python 3.8. For a more extensive explanation on the clustering procedure see Supplementary Information B.

### 2.6 HPV typing

Immunohistochemical analysis was performed for p16^INK4A^. Strong and diffuse nuclear and cytoplasmic immunostaining in more than 70% of the tumor cells was considered as p16 positive [17], [18], [19]. If HPV status was available, this was used instead of the p16 staining due to its lower false positive rate.

### 2.7 Response assessment

Patients were followed by the HN multidisciplinary team and response evaluation was performed by clinical examination and MR imaging, if indicated. Follow-up visits were bi-monthly for the first year following radiotherapy. Progressive disease (PD) within one year was defined as local disease, regional disease, distant metastasis, or any combination thereof present within one year after the end of radiotherapy. Complete response (CR) was defined as the absence of PD.

### 2.8 Statistical analysis

The average *ADC*, *f*, D*, *D*, *K*, and fractional cluster contribution were compared between HPV-positive and HPV-negative tumors using Wilcoxon rank sum tests. The parameter values (*f*, D*, *D*, *K*) per cluster were compared using Kruskal Wallis and Dunn’s post hoc tests. The average *ADC*, *f*, D*, *D*, *K,* fractional cluster contribution and response within one year were compared with Wilcoxon rank sum tests in subgroup analyses of HPV-negative and HPV-positive patients separately. To investigate a possible confounding effect of T and N stage on response, the correlation between tumor volume, T and N stage, and *ADC*, *f*, D*, *D*, and *K* was investigated using Spearman correlation (r_s_) in subgroup analysis of HPV-negative and HPV-positive patients separately. A p-value of p < 0.05 was considered statistically significant. No correction for multiple testing was used. All statistical analyses were carried out with the SciPy package (version 1.10.1) in python 3.8.

## 3 Results

### 3.1 Patient characteristics

Two patients had to be excluded due to insufficient signal at *b* = 1500 s/mm^2^. In total 36 patients remained, of which 18 were HPV-positive and 18 HPV-negative. The average GTV volume was 16.3 cc (range 0.9–106.7 cc). Table 1 shows the patient characteristics and response one year post (chemo-)radiotherapy per HPV status. Supplementary Table C1 shows the patient characteristics in the HPV-negative group per treatment outcome (CR or PD).

**Table 1:** Patient characteristics. Tumor staging was done according to TNM classification, edition 8.

	Total	HPV-positive	HPV-negative
N	36	18	18
Age [years] (mean ± SD)	62 ± 8	61 ± 7	62 ± 9
Sex			
Male	26	13	13
Female	10	5	5
T Stage			
T1-2	22	13	9
T3-4	14	5	9
N stage			
N0	13	4	9
N+	23	14	9
M stage			
M0	36	18	18
M+	0	0	0
Tumor volume [cc]	16 ± 22	13 ± 12	20 ± 28
Smoking at start RT			
Yes	20	7	13
No	16	11	5
Never smokers	5	5	0
Former smokers	11	6	5
Radiotherapy			
Photons	26	14	12
5 fr/week	14	9	5
6 fr/week	12	5	7
Protons	10	4	6
5 fr/week	6	4	2
6 fr/week	4	0	4
Chemotherapy			
Yes	26	15	11
Cisplatin	17	12	5
Cetuximab	9	3	6
No	10	3	7
Response one-year post-RT			
Complete response	28	16	12
Progressive disease	8	2	6
Local failure	5	2	3
Regional failure	3	1	2
Distant metastasis	3	0	3

### 3.2 Differences between HPV-positive and HPV-negative patients

**Fig. 2:**
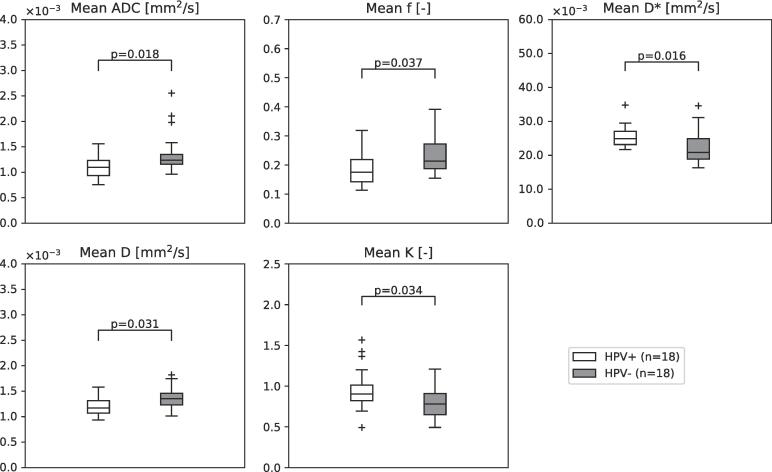
Boxplots of the ADC and the NG-IVIM parameters in which the white boxplot depicts parameter values for the HPV-positive patients and the grey boxplot for the HPV-negative patients. The horizontal line represents the median and the box represents the 25th to 75th percentile.

Fig. 2 shows boxplots of the distributions of the *ADC* and NG-IVIM parameters over the patients stratified by HPV status. In the HPV-negative group, *ADC* and the NG-IVIM parameters *D* and *f* were significantly higher than in the HPV-positive group: for *ADC* 1.4 ± 0.4·10^-3^ mm^2^/s versus 1.1 ± 0.2·10^-3^ mm^2^/s (p = 0.018); for *D* 1.4 ± 0.2·10^-3^ mm^2^/s versus 1.2 ± 0.2·10^-3^ mm^2^/s (p = 0.031), for *f* 0.24 ± 0.08 versus 0.19 ± 0.06 (p = 0.037). The D* and *K* were significantly lower in the HPV-negative group compared to the HPV-positive group: for D* 2.3 ± 0.5·10^-2^ mm^2^/s versus 2.5 ± 0.3·10^-2^ mm^2^/s (p = 0.016), for *K* 0.8 ± 0.2 versus 1.0 ± 0.3 (p = 0.034).

### 3.3 Intra-tumor regions

**Fig. 3:**
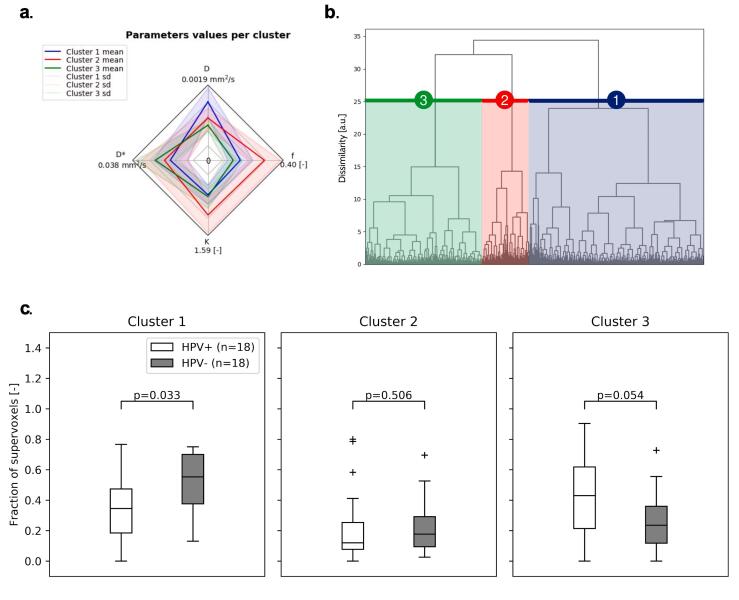
(a) A spider web plot is shown with the average value of each parameter for the three clusters. The center of the spider web plot represents the value 0. (b) The clustering tree is shown with the corresponding cluster numbers referred to in (c). (c) Boxplots of the fractional contribution of each of the clusters for HPV-positive and HPV-negative patients. The p-values are not corrected for multiple testing. The horizontal line represents the median and the box represents the 25th to 75th percentile.

In order to assess differences in intra-tumor regions between HPV-positive and HPV-negative patients, the tumors were divided in 4 to 483 supervoxels per tumor depending on tumor size, with a mean of 75 supervoxels per tumor. The optimal number of clusters according to the Calinski-Harabasz index was three. The average parameter values of these three clusters are schematically depicted in Fig. 3a. For all parameters (*f*, D*, *D,* and *K*), the parameter values were significantly different between all clusters (p < 0.001).

Cluster one showed a significantly higher contribution in HPV-negative tumors compared to HPV-positive tumors (p = 0.033), while cluster three showed a trend towards lower contribution in HPV-negative tumors compared to HPV-positive tumors (p = 0.054) (Fig. 3c).

### 3.4 Tumor response, HPV status, and conventional DWI and NG-IVIM DWI parameters

**Fig. 4:**
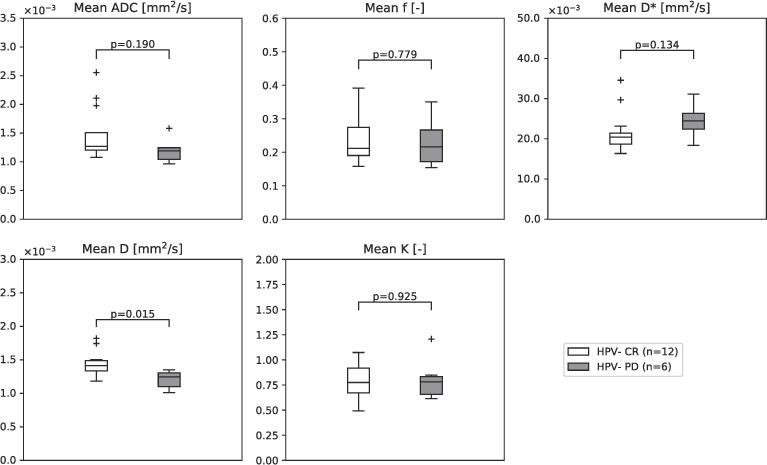
Boxplots of the ADC and the NG-IVIM parameters; white boxplot depicts parameter values for the HPV-negative patients with a complete response (CR) and the grey boxplot for the HPV-negative patients with progressive disease (PD). The horizontal line represents the median and the box represents the 25th to 75th percentile.

Due to the rare occurrence of PD in HPV-positive patients within one year (2 out of 18 patients), subgroup analysis was only performed for the HPV-negative patient group, in which 6 out 18 had PD within one year after treatment. The mean *D* was significantly lower in HPV-negative patients with PD at 1.2 ± 0.1·10^-3^ mm^2^/s compared to HPV-negative patients with a CR at 1.4 ± 0.2·10^-3^ mm^2^/s (p = 0.015). *ADC*, *f*, D* and *K* did not show significant differences between CR and PD (Fig. 4). No significant correlation between T stage or N stage and the DWI parameters *ADC*, *D*, *f*, D* and *K* was found in the HPV-negative subgroup (p-value > 0.05 and r_s_ ranged from −0.45 to 0.30). This suggests that T and N stage are not confounding factors for the relation between DWI parameters and response. For tumor volume, a significant correlation was found with *f* (r_s_ = −0.61, p = 0.007), but not for the other parameters. Therefore, this suggests that tumor volume is not a confounding factor for the relation between ADC, D*, D and *K* and response.

**Fig. 5:**
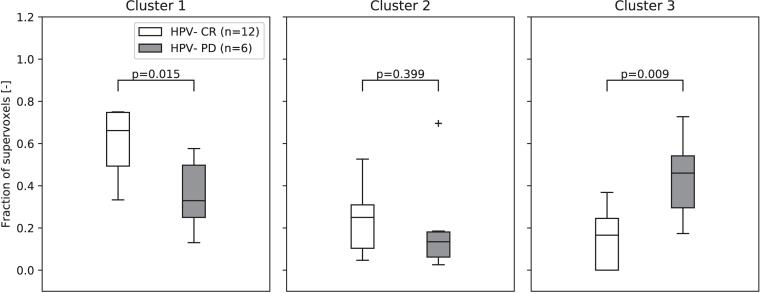
Boxplots of the fractional contribution of each of the clusters for HPV-negative complete responders (CR) and HPV-negative patients with progressive disease (PD) within one year. The p-values are not corrected for multiple testing. The horizontal line represents the median and the box represents the 25th to 75th percentile.

Cluster one showed a significantly higher contribution in HPV-negative patients with a CR compared to progressive disease (p = 0.015), while cluster three showed a significantly lower contribution in HPV-negative patients with a CR compared to PD (p = 0.009) (Fig. 5).

## 4 Discussion

In this study, we describe the first clinical application of our recently optimized NG-IVIM acquisition for a group of 36 oropharyngeal tumor patients. NG-IVIM enables simultaneous assessment of *inter*-cellular diffusion (similar to conventional DWI), microvascular perfusion and *intra*-cellular diffusion.

We found that the *ADC* and all NG-IVIM parameters were related to HPV status. HPV-negative patients had a higher *ADC* and NG-IVIM parameter *D* than HPV-positive patients, which is in line with existing literature [4], [9], [20], [21], [22], [23], [24], [25], [26], [27], [28], [29], [30], [31]. Since HPV-negative patients in general have a worse response to treatment than HPV-positive patients, that finding by itself would suggest that a high *ADC* is related to a poorer response, which has been reported also before in studies about pre-treatment DWI that did not correct for HPV status [1], [32], [33]. However, in our cohort of HPV-negative patients, the opposite correlation was found, namely that a lower NG-IVIM parameter *D* was related to poorer response. No relation between *ADC* and response was observed. In other words, our results could suggest that *ADC* is a surrogate for HPV status and is not related to response, while NG-IVIM parameter *D* was related to response in the HPV-negative group. This could be a concrete indication of the added value of NG-IVIM compared to conventional DWI. It also implies that NG-IVIM response analyses should be performed separately for HPV-positive and HPV-negative patients.

Martens et al. [3] did correct for HPV status using a multimodality CoxBoost regression analysis, but none of the pre-treatment IVIM parameters nor *ADC* were significant predictors for locoregional failure. Yet, when assessing overall survival, Martens et al. [3] did find both pre-treatment high mean *ADC* and low D* as predictive factors for poor overall survival, while Ravanelli et al. [2] did not find any correlation between pre-treatment *ADC* and overall survival when analyzing the HPV-positive and negative group separately. Similarly, Connor et al. [30] did not find a correlation between pre-treatment *ADC* and disease free survival after 2 years in the subgroup containing HPV-positive OPSCC only, nor in the subgroup of the other head and neck carcinomas (including HPV-negative OPSCC). Therefore, further research is needed to determine the prognostic value of DWI parameters within HPV subgroups.

The range of b-values can influence the calculated DWI values. For example, the *ADC* may be biased substantially depending on whether b-values in the perfusion range (0–200 s/mm^2^) and restricted diffusion range (>800 s/mm^2^) are used or not. This could explain the different ADC values in literature, the insignificant difference in *ADC* between HPV-negative CR and PD, and the stronger difference in *ADC* between HPV-positive and HPV-negative tumors than *D*. Here, we used a b-value set that was previously optimized for NG-IVIM as a step towards standardization [7].

The higher *ADC* and *D* in HPV-negative tumors compared to HPV-positive tumors could be due to the fact that HPV-negative tumors tend to have variable cellularity and a high stromal content, whereas HPV-positive tumors tend to have back-to-back densely packed cells and less tumor stromal component [22].

NG-IVIM parameter *K* quantifies the non-Gaussian diffusion behavior of water molecules when diffusion is restricted by cell membranes or other microstructural components [6]. This means that tissue with smaller cells has a higher *K*. The lower average *K* found in HPV-negative tumors compared to HPV-positive tumors could be caused by the fact that HPV-positive tumors have more cells with basaloid appearance (which are characterized by smaller cells) and more infiltration of lymphocytes [34]. This highlights the benefit of extending the conventional DWI model not only to the IVIM DWI model, but to the NG-IVIM DWI model to incorporate the non-Gaussian diffusion behavior of water molecules.

We also found differences in *f* and D* between HPV-positive and negative OPSCC. The higher average *f* and lower average D* in HPV-negative tumors compared to HPV-positive tumors suggest that even though there is more blood volume, the blood velocity is lower in HPV-negative tumors. These trends in perfusion parameters might suggest that HPV-negative OPSCC have a less functional vasculature. This hypothesis is supported by the study of Hanns et al. [35] that showed a lower density of neo-blood vessels, more hypoxic tumor areas, and higher mRNA expression of hypoxia-responsive genes in HPV-negative tumors compared to HPV-positive HN tumors. However, Vidiri et al. [27] did not find any significant differences in *f* and D* for HPV-negative versus positive OPSCC, and contradicting literature can be found about the vascularization of HPV-negative and positive tumors [35], [36].

In addition to the average parameter value, intra-tumor heterogeneity was investigated by analyzing regions with similar NG-IVIM parameter values, using unsupervised clustering. One cluster had a significantly higher presence in HPV-negative tumors and one cluster had a higher, albeit not significantly, presence in HPV-positive tumors. This suggests that HPV-negative tumors often have regions with a relatively high *D* (related to a high amount of stroma) and HPV-positive tumors often have regions with a relatively high D* (related to high blood velocity).

DWI in the HN is prone to motion. While most head motion is mitigated as patients are scanned in the immobilization mask, misalignment between the *b* = 0 s/mm^2^ and higher b-values due to swallowing and/or coughing may occur. In general, swallowing/coughing artifacts affect only one b-value, so unless a patient was coughing or swallowing excessively, the effect on the parameter values will be minimal.

In this study, we focused on the possible value of pre-treatment DWI for response assessment of the primary tumor. Several prior studies indicated that lymph node analysis [37] and mid-treatment DWI obtaining DWI during treatment [38], [39], [40], [41] could also be interesting for response assessment.

This study has some limitations. First, we used p16 status as a proxy for HPV status. However, p16 has a false positive rate of around 5–20% [42]. Therefore, it is likely that some patients defined as HPV-positive in this study were false positives. Second, due to the small sample size, relatively short follow-up and the single-center nature of the study, more research should be performed to ensure the results are generalizable. Moreover, due to the relatively small sample size, we did not correct for multiple testing. Finally, a limitation of unsupervised clustering is that adding or removing patients could result in slightly different clusters than presented here.

In conclusion, we found differences in *ADC* as well as all NG-IVIM parameters and in cluster analyses between HPV-positive and HPV-negative OPSCC. In a subgroup analyses of only HPV-negative patients, we found that *D* negatively correlated with progressive disease, which contradicts current literature relating *ADC* and *D* to progressive disease without correcting for HPV status. This suggests that *ADC* and *D* estimated in those studies could potentially be a surrogate for HPV status instead of a response predictor. Therefore, HPV status should be corrected for when assessing the predictive value of DWI. We found no correlation between response and *ADC*, indicating the potential added value of the more elaborate NG-IVIM model compared to conventional DWI.

## CRediT authorship contribution statement

**Nienke D. Sijtsema:** Conceptualization, Methodology, Software, Formal analysis, Investigation, Visualization, Writing – original draft, Writing – review & editing. **Iris Lauwers:** Conceptualization, Methodology, Software, Formal analysis, Investigation, Visualization, Writing – original draft, Writing – review & editing. **Gerda M. Verduijn:** Conceptualization, Methodology, Data curation, Writing – review & editing. **Mischa S. Hoogeman:** Supervision, Writing – review & editing. **Dirk H.J. Poot:** Resources, Supervision, Writing – review & editing. **Juan A. Hernandez-Tamames:** Supervision, Writing – review & editing. **Aad van der Lugt:** Conceptualization, Writing – review & editing. **Marta E. Capala:** Conceptualization, Methodology, Data curation, Writing – review & editing. **Steven F. Petit:** Conceptualization, Methodology, Funding acquisition, Supervision, Writing – review & editing.

## Declaration of Competing Interest

The authors declare the following financial interests/personal relationships which may be considered as potential competing interests: This work was funded by a research grant from Elekta AB (Stockholm, Sweden) and a research grant from The Dutch Cancer Society (KWF 2019-12141). Erasmus MC Cancer Institute also has a research collaboration with Accuray Inc (Sunnyvale, CA, USA) and Varian, a Siemens Healthineers Company (Palo Alto, CA, USA).
