# Supplementary material for: Relating pre-treatment non-Gaussian intravoxel incoherent motion diffusion-weighted imaging to human papillomavirus status and response in oropharyngeal carcinoma
Source: Phys Imaging Radiat Oncol. 2024 Apr 4;30:100574. doi: 10.1016/j.phro.2024.100574 (PMC11021835; doi:10.1016/j.phro.2024.100574)
Supplement: Supplementary A [file mmc1.docx]

**Supplementary Information A: NG-IVIM and ADC DWI fitting**

For conventional DWI, voxel-wise least square fitting was done with the mono-exponential model (Eq. A.1) and for NG-IVIM DWI according to the NG-IVIM model (Eq. A.2).

$S_{i}=S_{0}e^{-b_{i}ADC}$ (Eq. A.1)

In Eq. A.1 *S_i_* is the measured signal intensity at the corresponding b-value *b_i_,* *S_0_* the signal intensity at b-value of 0 s/mm^2^ and *ADC* is the apparent diffusion coefficient.

$S_{i}=S_{0}\left( \left( 1-f \right) e^{-b_{i}D+{\frac{1}{6}\left( b_{i}D \right)}^{2}K}+fe^{-b_{i}D} \right)$ (Eq. A.2)

In Eq. A.2 *S_i_* is the measured signal intensity at the corresponding b-value *b_i_*, *S_0_* the signal intensity at b-value of 0 s/mm^2^, *D* the diffusion coefficient, *f* the perfusion fraction, *D** the pseudo-diffusion coefficient, and *K* the kurtosis.

All fitting was done within the tumor volume using all b-values except b=0 s/mm^2^ with an in-house fitting algorithm employing a multiple starting point method written in MATLAB (MathWorks, Natick, MA, USA). For each voxel 1000 starting points were chosen between 0.25·10^-3^ and 3.41·10^-3^ mm^2^/s for *ADC* and *D*, 0.09 and 0.42 for *f*, 6.29·10^-3^ and 23.39·10^-3^ mm^2^/s for *D** and between 0.1 and 3 for *K* according to a Halton sequence [1]. These starting points form a set of low-discrepancy, pseudo random vectors, and the ranges were chosen based on previous work [2] and visual inspection of the fit results. To avoid non-physiological results and extreme outliers, the following fitting constraints were used: 0 to 20·10^-3^ mm^2^/s for *ADC* and *D*, 0 to 1 for *f*, 0 to 200·10^-3^ mm^2^/s for *D**, and 0 to 5 for *K.*

**References**

[1] Wang X, Hickernell FJ. Randomized Halton sequences. Math Comput Model. 2000;32:887-99.

[2] Sijtsema ND, Petit SF, Poot DHJ, Verduijn GM, van der Lugt A, Hoogeman MS, et al. An optimal acquisition and post-processing pipeline for hybrid IVIM-DKI in head and neck. Magn Reson Med. 2021;85:777-89.
