# Supplementary material for: Relating pre-treatment non-Gaussian intravoxel incoherent motion diffusion-weighted imaging to human papillomavirus status and response in oropharyngeal carcinoma
Source: Phys Imaging Radiat Oncol. 2024 Apr 4;30:100574. doi: 10.1016/j.phro.2024.100574 (PMC11021835; doi:10.1016/j.phro.2024.100574)
Supplement: Supplementary B [file mmc2.docx]

**Supplementary Information B: Assessing intra-tumor regions**

**Filtering out noise**

First, noise was filtered out by creating supervoxels using the Simple Linear Iterative Clustering (SLIC) algorithm [1] for each tumor separately. Supervoxels are combinations of several voxels that are located close together and have similar image intensity across multiple parameter maps. To ensure equal weighting of the parameters (*f, D*, D,* and *K*), each parameter was normalized separately before running the supervoxel algorithm. The normalization was based on the average of the mean parameter value per GTV ($\bar{p}$) and the average of the standard deviation per GTV ($\bar{p_{std}}$) over all patients according to Eq. B.1 in which, $p_{normalized}$ and $p$ are the normalized and not normalized parameter values of a voxel respectively. Within the SLIC supervoxel algorithm, the average size was set to 50 ± 10 voxels and the compactness to 20.

$p_{normalized}=\frac{p-\bar{p}}{\bar{p_{std}}}$ (Eq. B.1)

**Clustering algorithm**

After creating supervoxels, the average normalized parameter values were calculated per supervoxel as input for the clustering algorithm. The resulting supervoxels of all patients were combined into one dataset. The dataset was clustered using an agglomerative Ward clustering algorithm based on the average normalized parameter values [2]. This algorithm starts by assigning a cluster to each individual supervoxel. Subsequently, an iterative process started in which two clusters were combined into new clusters until only one cluster was left. The Ward method was used to decide which clusters were combined per iteration step. This entailed that the variance per cluster was calculated as well as the variance of each possible newly combined cluster. The newly combined cluster causing the smallest rise in total variance was selected.

**Selecting the optimal number of clusters**

After creating this cluster hierarchy, we had to choose which number of clusters would be optimal for our analysis. When using too many clusters, clusters can be very similar and might not relevantly differ from each other. On the other hand, using too few clusters can cause high intra-cluster variances and thereby erase relevant differences. Therefore, it was important to have a trade-off between low intra-cluster and high inter-cluster variation. The Calinski-Harabasz index is a measure that penalizes intra-cluster variability and rewards inter-cluster variability (Eq. B.2) [3].

$CH= \left[ \frac{\sum_{k=1}^{K} {n_{k}\left\| c_{k}-c \right\|}^{2}}{K-1} \right]/\left[ \frac{\sum_{k=1}^{K} \sum_{i=1}^{n_{k}} \left\| d_{i}-c_{k} \right\|^{2}}{N-K} \right]$ (Eq. B.2)

In this formula, K is the number of clusters on the dataset D = [$d_{1},d_{2},\ldots,d_{N}$]. n_k_, and c_k_ are the number of points and centroid of the k^th^ cluster respectively. c is the global centroid and N is the total number of data point. The cluster number with the highest Calinski-Harabasz index was selected. In our case, this was three (Figure B.1).


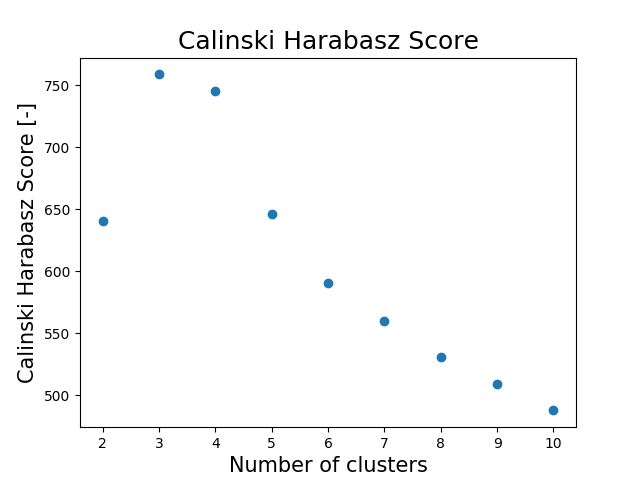


Figure B.1: An overview of the Calinski-Harabasz index for the different number of clusters. The higher the Calinski-Harabasz the better the clusters describe the variation in the data in terms of minimal intra-cluster variation and maximum inter-cluster variation.

**Cluster prevalence**

Per tumor, the prevalence of each cluster was calculated by dividing the number of supervoxels belonging to a cluster by the total number of supervoxels of the tumor (the fractional contribution).

**References**

[1] Achanta R, Shaji A, Smith K, Lucchi A, Fua P, Süsstrunk S. SLIC Superpixels Compared to State-of-the-Art Superpixel Methods. IEEE Transactions on pattern analysis and machine intelligence. 2012;34:2274-81.

[2] Ward JH. Hierarchical Grouping to Optimize an Objective Function. Journal of the American Statistical Association. 1963;58:236-44.

[3] Caliński T, Harabasz J. A dendrite method for cluster analysis. Communications in Statistics. 1974;3:1-27.
