# Supplementary material for: Relating pre-treatment non-Gaussian intravoxel incoherent motion diffusion-weighted imaging to human papillomavirus status and response in oropharyngeal carcinoma
Source: Phys Imaging Radiat Oncol. 2024 Apr 4;30:100574. doi: 10.1016/j.phro.2024.100574 (PMC11021835; doi:10.1016/j.phro.2024.100574)
Supplement: Supplementary C [file mmc3.docx]

Table C.1. Patient characteristics of HPV-negative patients per response group. Tumor staging was done according to TNM classification, edition 8.

|  | HPV negative | Complete response | Progressive disease |
| --- | --- | --- | --- |
| N | 18 | 12 | 6 |
| Age [years] (mean ± SD) | 62±9 | 60±8 | 67±8 |
| Sex |  |  |  |
| Male | 13 | 8 | 5 |
| Female | 5 | 4 | 1 |
| T Stage |  |  |  |
| T1-2 | 9 | 7 | 2 |
| T3-4 | 9 | 5 | 4 |
| N stage |  |  |  |
| N0 | 9 | 9 | 0 |
| N+ | 9 | 3 | 6 |
| M stage |  |  |  |
| M0 | 18 | 12 | 6 |
| M+ | 0 | 0 | 0 |
| Tumor volume [cc] | 20±28 | 18±22 | 25±36 |
| Smoking at start RT |  |  |  |
| Yes | 13 | 9 | 4 |
| No | 5 | 3 | 2 |
| Never smokers | 0 | 0 | 0 |
| Former smokers | 5 | 3 | 2 |
| Radiotherapy |  |  |  |
| Photons | 12 | 11 | 1 |
| 5 fr/week | 5 | 4 | 1 |
| 6 fr/week | 7 | 7 | 0 |
| Protons | 6 | 1 | 5 |
| 5 fr/week | 2 | 0 | 2 |
| 6 fr/week | 4 | 1 | 3 |
| Chemotherapy |  |  |  |
| Yes | 11 | 7 | 4 |
| Cisplatin | 5 | 4 | 1 |
| Cetuximab | 6 | 3 | 3 |
| No | 7 | 5 | 2 |
